# Treatment of Low Back Pain with a Digital Multidisciplinary Pain Treatment App: Short-Term Results

**DOI:** 10.2196/rehab.9032

**Published:** 2017-12-04

**Authors:** Stephan Huber, Janosch A Priebe, Kaja-Maria Baumann, Anne Plidschun, Christine Schiessl, Thomas R Tölle

**Affiliations:** ^1^ Department of Neurology, Center for Interdisciplinary Pain Medicine Klinikum rechts der Isar Technical University of Munich Munich Germany; ^2^ Kaia Health Software Munich Germany

**Keywords:** lower back pain, app, mHealth, retrospective study, self-management

## Abstract

**Background:**

Even though modern concepts of disease management of unspecific low back pain (LBP) postulate active participation of patients, this strategy is difficult to adapt unless multidisciplinary pain therapy is applied. Recently, mobile health solutions have proven to be effective aides to foster self-management of many diseases.

**Objective:**

The objective of this paper was to report on the retrospective short-term results of a digital multidisciplinary pain app for the treatment of LBP.

**Methods:**

Kaia is a mobile app that digitalizes multidisciplinary pain treatment and is in the market as a medical product class I. For the current study, the data of anonymized Kaia users was retrospectively analyzed. User data were evaluated for 12 weeks regarding duration of use and effect on in-app user reported pain levels, using the numerical rating scale (NRS), depending on whether LBP was classified as acute, subacute, or chronic back pain according to current guidelines.

**Results:**

Data of 180 users were available. The mean age of the users was 33.9 years (SD 10.9). Pain levels decreased from baseline NRS 4.8 to 3.75 for all users at the end of the observation period. Users who completed 4, 8, or 12 weeks showed an even more pronounced decrease in pain level NRS (baseline 4.9 [SD 1.7] versus 3.6 [SD 1.5] at 4 weeks; baseline 4.7 [SD 1.8] versus 3.2 [SD [2.0] at 8 weeks; baseline 4.6 [SD 2.2] versus 2.6 [SD 2.0] at 12 weeks). In addition, subgroup analysis of acute, subacute, or chronic classification revealed no significant main effect of group (*P*>.30) on the reduction of pain. Conclusions: This retrospective study showed that in a pre-selected population of app users, an app digitalizing multidisciplinary rehabilitation for the self-management of LBP reduced user-reported pain levels significantly. The observed effect size was clinically relevant. Ongoing prospective randomized controlled trials (RCTs) will adjust for potential bias and selection effects.

**Conclusions:**

This retrospective study showed that in a pre-selected population of app users, an app digitalizing multidisciplinary rehabilitation for the self-management of LBP reduced user-reported pain levels significantly. The observed effect size was clinically relevant. Ongoing prospective RCTs will adjust for potential bias and selection effects.

## Introduction

In spite of recent developments in diagnosis and treatment of low back pain (LBP), the burden of disease for patients and health economy remains outstanding. LBP is not only the leading cause of years lived with disability globally, but shows a 1-month prevalence of about 30% of the global population. The vast majority of patients are affected by non-specific LBP rather than by back pain with a specific cause that can be targeted by a specific treatment [1].

Recently, treatment paradigms have shifted from a merely somatic disease concept of LBP towards a bio-psycho-social model; a more comprehensive approach that encompasses somatic findings as well as psychological and environmental factors. Current treatment of LBP in primary and secondary care is often limited to a monocausal somatic approach and thus disregards current guidelines [2]. Multidisciplinary pain treatment (MPT), a combined program comprising educational, physical, and psychological exercises, has been proven to be effective in the treatment of LBP with positive effects on pain level, functionality, and other outcomes parameters including quality of life [3]. As such, MPT is part of treatment recommendations for chronic LBP in a variety of international guidelines [4-6].

Multidisciplinary programs are comparably expensive and limited to specialized centers, which restricts their widespread use. Only recently, electronic health (eHealth) and mobile health (mHealth) Web apps have emerged as new treatment options for non-pharmacologic interventions in a variety of conditions [7]. Guidelines for the development of mHealth apps are under development and the rapidly progressing field awaits constant adjustments in structure, composition, and content [8,9]. Especially in chronic conditions, which require adequate strategies of self-management for optimal treatment results, mobile- or Web-based solutions show great potential and sometimes even more desirable outcomes than current—often pharmacologic—standard therapies [7].

Several mHealth or Web-based solutions have been designed for the self-management of LBP; however, only few of them have been subjected to prospective clinical trials [10]. Two recent reviews considered 9 and 6 clinical studies relevant, respectively [11,12]. However, all of them represented a vast variety of different approaches, many of them based on cognitive behavioral strategies. Due to the heterogeneity of the included interventions and primary endpoints, the authors found the evidence inconclusive [11]. The clinical standard of LBP treatment considers physical activity and activation [1,5,6]. Recent appraisals of commercially available apps revealed that the vast majority of apps available in app stores are not based on a scientific framework [13]. Surprisingly, physical activity was only included as a key component in 1 app and study [12].

Here, we reported on the efficacy of an LBP app that is based on a comprehensive multidisciplinary treatment concept, including patient education, video-guided physiotherapy, and mindfulness training. The content of the app is in line with current German guidelines for the management of LBP [5]. The study investigated the in-app reported pain levels of users in their pain diaries to elucidate the development of pain levels over a period of 3 months after download of the app.

## Methods

### Study Design and Users

The study was designed as a retrospective analysis of the user database of Kaia. All users agreed to the collection of data presented in this publication by signing the terms and conditions for use of Kaia. All data used for the study were anonymized before submission to the Technical University of Munich for statistical analysis.

The study cohort was recruited via online channels (Facebook, Google Ads, company homepage) in Germany, Austria, and Switzerland. The criteria for participation were age 18 years and older, declaration of medical treatment of back pain, no history of indicators for specific causes of back pain (red flags), and sufficient level of physical fitness (self-report). The study sample consisted of all users in the user database of the company fulfilling the inclusion criteria.

Users included in the study had to be users of the Pro version, as non-Pro users are limited to 1 week of usage only. Only subscribers before March 2017 were included. The Institutional Ethic Committee of the Medical Faculty of the Technische Universität München approved the study design (study number 273-17s).

### Data Collection

All data analyzed in this study were entered by app users as part of their self-test or in-app diaries and stored on company servers in Frankfurt, Germany. Only anonymized data were extracted from the user database via reporting criteria and no personal data were submitted for scientific evaluation. The data protection officer of the University Hospital of the Technische Universität München approved the concept for protection of personal data of the current study.

### Statistical Analysis

Primary analysis referred to the comparison of baseline pain levels and the pain levels on the last day of use. For this purpose, mean baseline pain levels and mean last day of use pain levels were subjected to a paired-sample *t* test. In addition, for the purpose of investigating if completing the program (12 weeks) is advantageous compared to quitting the program at an earlier point of time, the following tests were conducted: (1) baseline pain level and pain level after 12 weeks were compared for those users completing the program using the paired-sample *t* test and checked effect sizes for differences (completers versus all users); and (2) a between-subject *t* test was computed in order to compare final pain levels of the completers (12 weeks) and all users.

Secondary analyses were also performed. In order to investigate the development of pain levels over time, 3 paired-sample *t* tests (Bonferroni-corrected) were computed in order to compare the baseline pain level with the pain level after 4 weeks (test 1), the pain level after 8 weeks (test 2), and the pain level after 12 weeks (test 3).

Furthermore, in order to detect potential differences between subgroups with different durations of LBP, baseline pain ratings, as well as pain ratings after 4 weeks, 8 weeks, and 12 weeks of training were subjected to 3 separate split-plot analysis of variances (ANOVAs) with the 3-level between-factor duration of symptoms (less than 6 weeks [acute], versus 6 to 12 weeks [subacute], versus greater than 12 weeks [chronic]), and the 2-level within-factor time ANOVA 1 (baseline versus 4 weeks), ANOVA 2 (baseline versus 8 weeks), and ANOVA 3 (baseline versus 12 weeks).

### Overall Description of the App

Kaia (Kaia Health Software GmbH, Munich, Germany) is a multiplatform app for iOS, Android, and native Web solutions. Kaia came to market September 2016 and is classified as a medical product class I. It is available via the App Store (iOS), the Google Play Store, or as a native website. Download of the app is free, but to remain active in the app for longer than 7 days, and to unlock the full functionality, users need upgrade to the Pro version via an in-app purchase.

The Kaia program was available on a monthly subscription during the timeframe of the study at costs of €9.99/per month. Multimodal offline programs in Germany have costs ranging from €2500 to €5000 depending on duration and program structure.

After registration in the app, users performed a mandatory self-test. During the first stage, users confirmed that they were not suffering from any complaints that may be indicative of a potentially specific cause of pain (red flags). The potential hints for red flags in a patient's history that are included in the app were based on a corresponding list in the current German guidelines [5]. Furthermore, users were required to confirm that they had already visited a physician because of their LBP and that there was no contraindication for physiotherapy. The self-test furthermore assessed pain distribution, pain duration (acute LBP of less than 6 weeks, subacute LBP of 6 to 12 weeks, and chronic LBP of less than 12 weeks, based on German guidelines [5]), pain intensity, and overall fitness.

Depending on the results of this initial test, exercise regimen and content were tailored to the individual user from a pool of 120 exercises based on an algorithm.

Users recorded their levels of pain and sleep using numerical rating scales (NRSs) at the end of each day of therapy in a pain diary as a separate function of the app. Pain was recorded from 0 to 10 (worst imaginable pain) whereas sleep was recorded from 0 (worst imaginable sleep) to 10 (best imaginable sleep). User progress within the app from day to day of practice and the development of user-reported pain and sleep were constantly visible in a screen. There is also a chat function in the app that connects users to a coach (physiotherapist or sport scientist) for motivational and exercise-related questions.

### App Content

The Kaia app involves the following pillars: (1) back pain-specific education, (2) physiotherapy, and (3) mindfulness techniques. Daily content consists of all 3 pillars. The content for an individual patient is compiled and updated from day to day (or upon each login) from a large background of exercises and skills archived in the app. Depending on the patient´s status of knowledge, practice, and progress this is adapted from day to day. Each section is comprehensive as a stand-alone—there is no obligation to perform all 3 sections in a single session.

**Figure 1:**
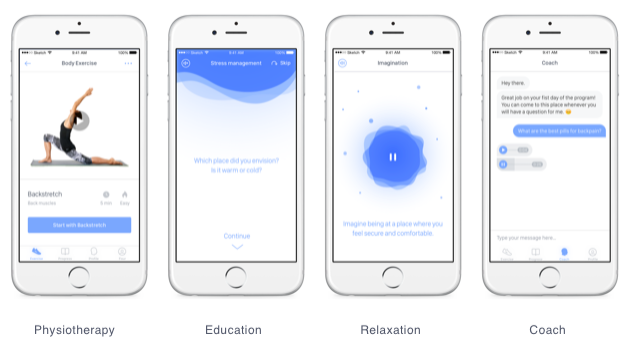
Examples of daily content from the app for each of the categories and design of the coach chat.

Content in the educational section covers a broad spectrum of general pain-related and back pain-specific education (Figure 1). There are over 30 different educational units in the app. Content is based on current German or international guidelines [5,6] and standard textbooks in the field. Educational content was authored by board-certified physicians with relevant expertise in the field of back pain (ie, neurology, orthopedic surgery, and pain medicine) or clinical psychologists with experience in pain psychotherapy.

The single exercises and the individual composition of exercises for every user per day (up to 5 exercises) were designed by physiotherapists of the Pain Center Technische Universität München according to guidelines and curricula of the German Pain Society. A pool of 145 exercises is subdivided into 5 classes (front side, lower back, upper back and shoulders, lateral muscles, and legs), and is individually applied in relation to the users body region with most pain. Furthermore, exercises within each class are ranked depending on exercise difficulty and strain. Depending on the self-test and ongoing user feedback, exercises are continuously adopted to the user’s fitness level.

Mindfulness and relaxation techniques are an integral part of multidisciplinary in- and outpatient LBP rehabilitation. The Kaia app contains units of breathing techniques, body scan, visualization, and progressive muscle relaxation. The value of the various techniques is explained in the education part of the app. Mindfulness content is generally broadcasted as audio content only.

## Results

### Sample Characteristics and Dropout of Users Over Time

Data of 180 users of the Pro version were available, of which 105 were female (58.3%, 105/180). The mean age of the users was 33.9 years (SD 10.9). Of the users, 25 (13.9%, 25/180) reported pain for less than 6 weeks, 23 (12.8%, 23/180) between 6 and 12 weeks, and 132 (73.3%, 132/180) patients reported pain for more than 12 weeks before starting the program.

**Figure 2:**
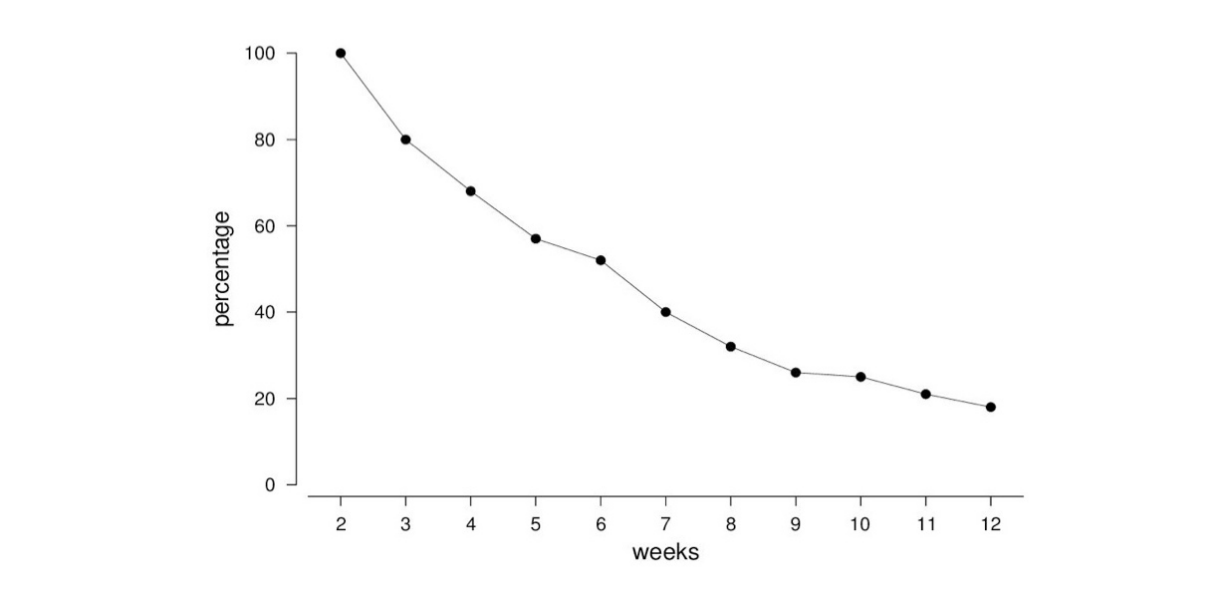
Development of user numbers over time.

As expected, there was a substantial dropout over time. After 4 weeks, the number of users decreased to 123 (68.3%, 123/180). After 8 and 12 weeks, 58 (32.2%, 58/180) and 32 (17.8%, 32/180) still participated in the program, respectively. The dropouts are illustrated in Figure 2.

### Development of User-Reported Pain Levels Until Last Reported Use

A significant reduction in pain level from the baseline (mean 4.80 [SD 1.95], median 5) to the last day of use (mean 3.75 [SD 1.76], median 4) was found (*t*_158_=6.21, *P*<.001, *d*=0.56). Moreover, in order to check if completers of the program (12 weeks, N=20) showed a better pain outcome, baseline pain levels (mean 4.60 [SD 2.21], median 4) and pain levels after 12 weeks (mean 2.60 [SD 1.98], median 3) of the completers were tested for differences. The paired-sample *t* test revealed a significant reduction in the pain level also in this group (*t*_19_=3.75, *P*=.001), with a bigger effect size compared to the overall comparison (*d*=0.95 versus *d*=0.56, see above). In addition, a between-group *t* test confirmed a significant better pain outcome for the program completers (2.60 versus 3.75) compared to all users, (*t*_177_=2.71, *P*=.007).

In addition, 2 ex-post-analyses were performed. Firstly, in order to analyze differences in baseline pain levels of completers and non-completers, a between-group *t* test was performed, which did not reveal significant differences (NRS 4.6 for completers versus 4.8 for non-completers, *t* less than 1). Secondly, to analyze whether users with lighter baseline pain levels had a different outcome than users with lower baseline pain levels, a median split was applied to the baseline pain level data and 2 paired-sample *t* tests were performed in order to compare baseline versus last day of use pain levels separately for users above and below median. A significant reduction in pain levels was found only in the above-median group (baseline 6.2 versus last day of use 4.2, –33%, *P*<.01). No significant baseline-last day of use differences were detected in the below-median group except a rather slight descriptive increase in pain levels (baseline 3.1 versus last day of use 3.3; +7%; *t* less than 1).

**Figure 3:**
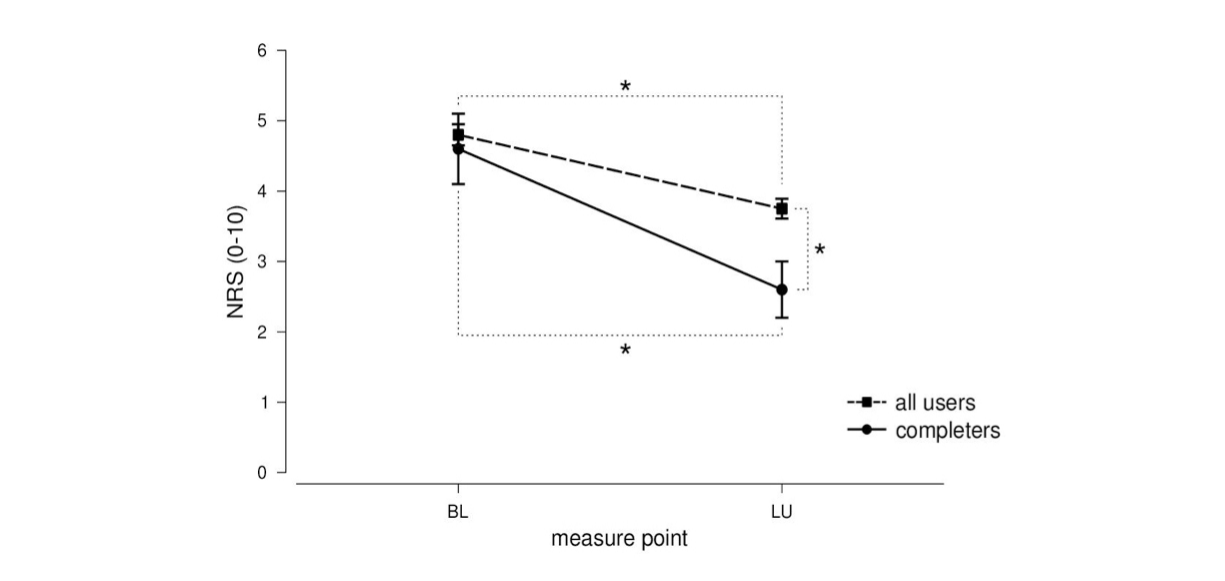
Mean (SE) baseline (BL) pain levels and pain levels of the day of the last use (LU) both for completers of the program (12 weeks) and all users. NRS: numerial rating scale.

These analyses revealed (1) a significant pain reduction over time through using the app; and (2) an even better pain outcome for completers of the program. All effects were of medium to large size (Figure 3).

### Development of Pain Levels Over Time

**Figure 4:**
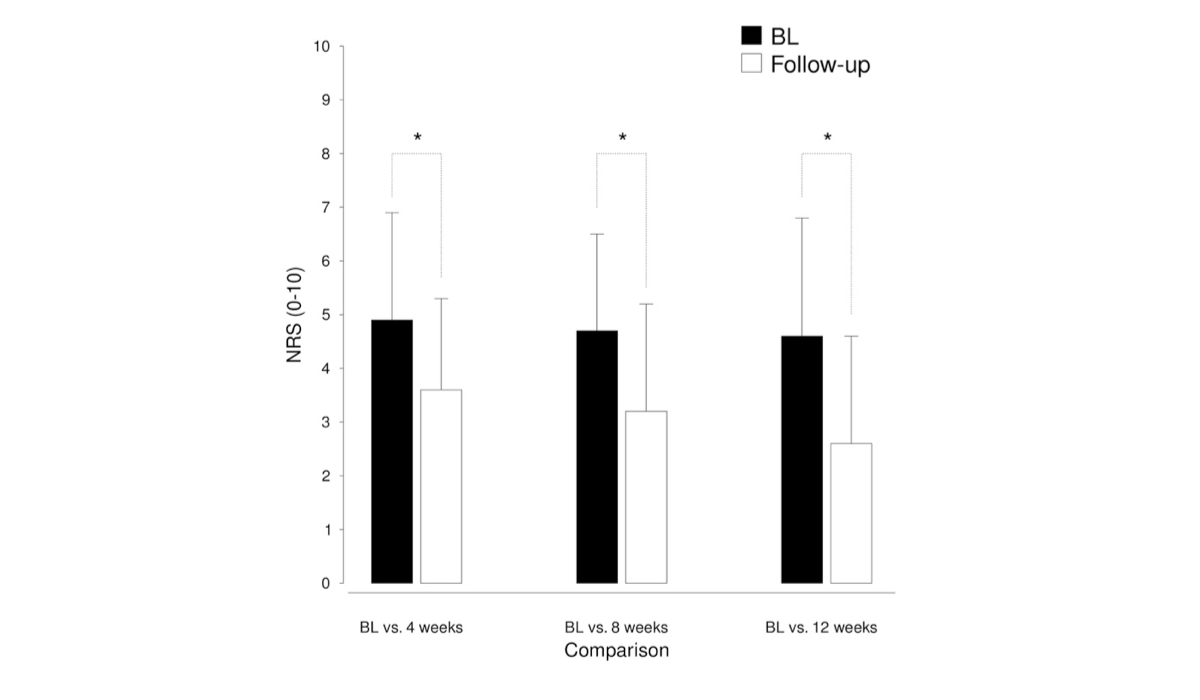
Development of mean (SD) pain levels both for the baseline (BL) and the 3 follow-up measures (4 weeks, N=71; 8 weeks, N=30; 12 weeks, N=20).

As the previous analysis revealed that users who remained in the app for 3 months had lower NRS scores at last use than users who quit at earlier points in time, we analyzed whether the decrease in NRS levels was larger over time using 3 follow-up measures (4, 8, and 12 weeks of use). The analysis revealed significant reductions in pain levels in all 3 follow-up measures relative to baseline: baseline versus 4 weeks follow-up (*t*_70_=6.10, *P*<.001, *d*=0.84), baseline versus 8 weeks follow-up (*t*_29_=3.64, *P*=.001, *d*=0.76), and baseline versus 12 weeks follow-up (*t*_19_=3.75, *P*=.001, *d*=0.95). The results of this analysis are depicted in Figure 4.

**Figure 5:**
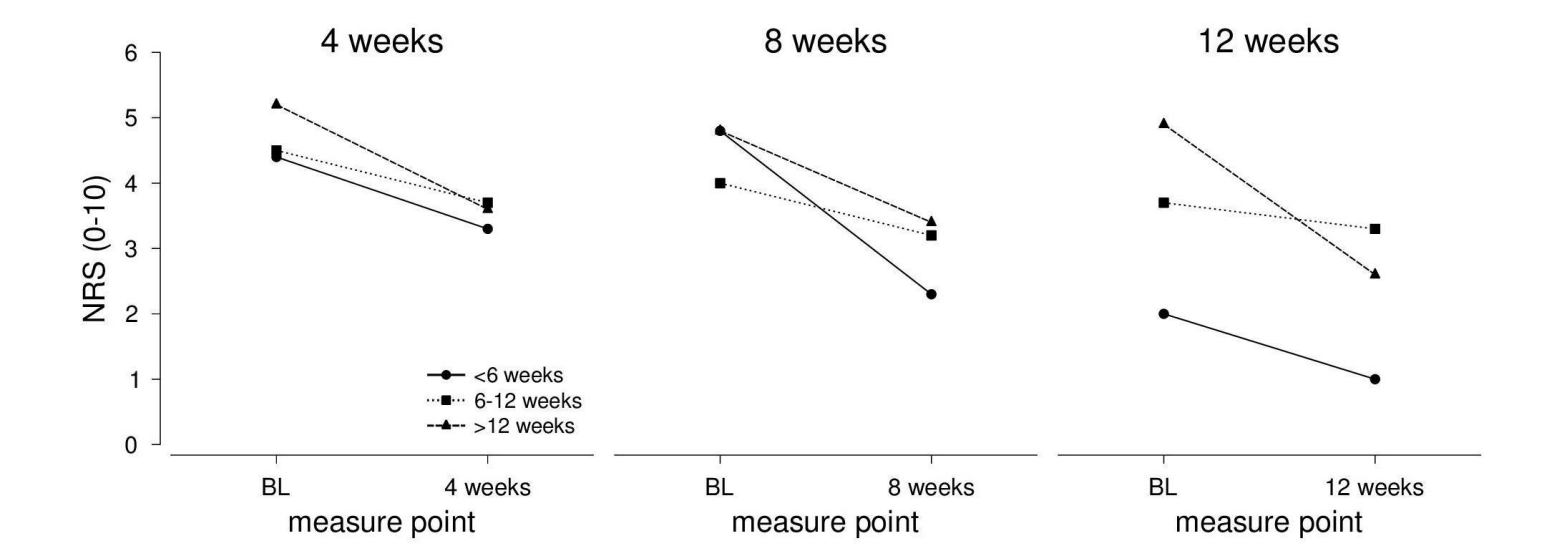
Development of pain levels over time (baseline versus 4, 8, and 12 weeks) for the 3 chronification groups (stratified by duration of their complaints). Less than 6 weeks: 4 weeks, N=12; 8 weeks, N=4; 12 weeks, N=1. Between 6 and 12 weeks: 4 weeks, N=10; 8 weeks, N=4; 12 weeks, N=3. More than 12 weeks: 4 weeks, N=49; 8 weeks, N=21; 12 weeks, N=16.

Pain level was reduced during app use regardless of the anamnestic duration of complaints. The duration of complaints was further analyzed whether the duration, as classified in the self-test (acute versus subacute versus chronic LBP), determined the pain reduction over time (Figure 5).

The ANOVAs for the pain ratings after 4 weeks and 8 weeks both revealed significant main effects of time (*F*_1,68_=17.28, *P*<.001, *η*= 0.203; *F*_1,27_=8.99, *P*=.006, *η*= 0.250), while there was no main effect of time in the ANOVA for the pain ratings after 12 weeks (*F*_1,17_=1.74, *P*=.205, *η*= 0.093). In addition, no significant main effect of group and no significant interaction of group and time were found in any of the ANOVAs (all *P* values less than .30). Taken together, an overall pain reduction was found in each of the groups after 4 weeks and after 8 weeks, but not after 12 weeks, suggesting that the effect of app use was equally effective regardless of the duration of complaints at start.

## Discussion

### Principal Findings

The application of a digital multidisciplinary back pain app reduced pain ratings in patients with LBP. The retrospective analysis of user data revealed stable pain reduction, independently of the duration of back pain (acute, subacute, chronic LBP) and demonstrated an increased level of pain reduction in relation to the duration of app application. Thus, the treatment of back pain can potentially be complemented with self-management via a digitalized version of a multidisciplinary biopsychosocial rehabilitation program. However due to its limitations, this retrospective set of data should only serve as a first pilot study and the effects should be confirmed with further prospective trials.

Recently, a large randomized controlled trial (RCT) of a mobile Web app by Irvine et al reported a significant decrease in pain burden following long-term use of a medical app [10]. And yet, another recent study confirmed that non-supervised exercise exerted a beneficial effect on pain levels and muscle strength as compared to patients on a wait-list [15]. Thus, the finding that self-management of LBP with an app reduced user reported pain levels fits well with these earlier observations and published data.

Recent reviews have not yet found conclusive evidence for the beneficial effects of digital solutions to support self-management of back pain [11,12]. This ambiguous view might be due to limited controlled trials [11,12]. The differences in the underlying concepts and also in the design of the interventions make it especially hard to generalize from results with one app to another. Of note, not one app explicitly based on a multidisciplinary setting was reported in these publications. Furthermore, this retrospective analysis did not reliably determine which section of the Kaia app (pain education, physiotherapy, or mindfulness training) was the key factor for pain improvement. The analysis of user log files and detailed feedback analysis (via path analyses) may help answer this question in future studies.

While apps utilized in clinical investigations related to back pain cannot be found in app stores, the ones found in app stores have not been applied in clinical investigations. It was also found that retrospective data for solutions was unavailable in app stores [13]. Thus, this current retrospective analysis is one of the first to bridge the gap between commercialized support interventions for back pain and scientific evaluation.

The dropout rate over 12 weeks was high. Of note, this is an early report and other similar publications also reported significant dropouts over time in digital interventions for self-management of musculoskeletal conditions while showing reduced pain levels in users still engaging in the app [16]. Future design of the app has addressed users’ feedback and included reminders like emails and push mails. Increasing interaction between patients and the app and personnel has been shown to contribute to user engagement of pain patients in a recent study [17].

The clinical potential of mHealth and eHealth to support the patient´s self-management and adoption of new behavioral patterns is not in question. This is underlined by increasing evidence and positive connotations in numerous disease conditions [7]. However, searches for apps in app stores that are intentionally designed for the self-management of pain often present with uncertain validity of content and are missing in scientific framework [13,18]. Establishing digital solutions based on current clinical concepts for self-management of LBP seems to be highly desirable, especially when considering that many patients seek online advice and support of self-management. The quality of content on self-management for back pain does not to reflect current medical knowledge for back pain treatment, as evidenced in several studies [19,20]. And, seemingly, the concept of self-management and active self-involvement in rehabilitation of pain has not reached a substantial level of awareness in patients suffering from LBP [21]. This preserves a passive attitude of patients and prevents new strategies of rehabilitation. On the other hand, this insight makes innovative methods, like apps, so important to spread the concept of self-management in LBP, especially given that the app is based on relevant concept and content.

Most previous online interventions in eHealth and mHealth apps for LBP have focused on cognitive behavioral therapy [10,11]. Only little scientific information is available for online interventions focused on exercise and relaxation techniques. However, trials with interventions focusing on the relevance and applicability of physical exercise and mindfulness are underway and currently ongoing [14,22,23].

### Limitations

Limitations of the current study arose from the uncontrolled, retrospective analysis. This did not allow adjustment of the reported decrease in pain levels for any potential spontaneous improvement of pain levels. The high rate of dropouts over time posed a significant limitation of the current study. Reasons for dropout are not known due to the study design, but across all users there was a substantial improvement in their pain levels from baseline to the last reported value, suggesting that overall users improved their pain levels during use of the app. However, whether this is caused by the app or spontaneous improvement will only be known after future RCTs. Furthermore, demographics of users included in the current study were not representative of the heterogeneous group of patients suffering from persistent back pain and represented only a selection of patients especially prone to profit from the digital intervention. This is valid since only limited data on the study collective are available due to the retrospective nature of the study—important information like the physical activity level at baseline was not known.

### Conclusions

The current retrospective study showed that in a pre-selected population of app users, an app digitalizing multidisciplinary rehabilitation for the self-management of LBP reduced user-reported pain levels significantly. The observed effect size was clinically relevant. Ongoing prospective RCTs will adjust for potential bias and selection effects.

## Abbreviations

ANOVA: analysis of variance
eHealth: electronic health
LBP: low back pain
mHealth: mobile health
MPT: multidisciplinary pain treatment
NRS: numerical rating scale
RCT: randomized controlled trial
